# Effects of Primary Healthcare Quality and Effectiveness on Hospitalization Indicators in Brazil

**DOI:** 10.3390/jmahp13020021

**Published:** 2025-05-09

**Authors:** Bruna Leão Freitas, Maria Luisa de Oliveira Collino Antiga, Flavia Mori Sarti

**Affiliations:** School of Arts, Sciences and Humanities, University of São Paulo, São Paulo 03828-000, Brazil

**Keywords:** healthcare management, health system, healthcare quality, healthcare effectiveness, patient satisfaction

## Abstract

Advances in primary healthcare coverage for the improvement in health outcomes at the population level comprise a major goal of public policies of health, particularly considering increases in hospitalization costs linked to chronic diseases in recent decades. Previous evidence shows the positive effects of access to primary healthcare on hospitalization indicators in high-income countries; however, there is a lack of literature on the subject in Latin American countries. Thus, the present study proposes a quantitative investigation on connections between primary healthcare quality and effectiveness in relation to hospitalization indicators, in addition to the identification of its effects on inequalities in hospitalizations in Brazil. The study was based on an empirical analysis of data from five cross-sectional surveys representative at the population level conducted by the Brazilian Institute for Geography and Statistics (IBGE) in 1998, 2003, 2008, 2013, and 2019. Information on the demographic, socioeconomic, and health characteristics of individuals compatible across surveys were included in the analyses, in addition to data on household and survey characteristics. The statistical analyses were based on the estimation of logistic regression models for the exploration of effects of primary healthcare quality and effectiveness on hospitalizations, inpatient days, and perception of quality of hospital care. Furthermore, the estimation of concentration indexes and their disaggregation allowed to verify trends and determinants of inequalities in hospitalization indicators in Brazil throughout the period. The results indicate that primary healthcare effectiveness is associated with the lower occurrence and frequency of hospitalizations, and a lower length of stay in hospitals. Primary healthcare quality was associated with the perception of higher quality of hospital care. Trends in hospitalization indicators showed reduction in inequalities towards low-income individuals from 1998 to 2013, and primary healthcare quality presented minor influence on inequalities in hospitalizations, inpatient days, and perception of quality of hospital care.

## 1. Introduction

Strategies based on health promotion and disease prevention have been implemented at the primary healthcare (PHC) level in diverse countries worldwide in recent decades, particularly due to the rise in prevalence, mortality, and hospitalization costs attributable to chronic diseases [1,2,3,4]. The acceleration of changes in population dynamics from the beginning of the 21st century onwards promotes alterations in epidemiological patterns at the global level, including increases in the prevalence of multimorbidity in diverse developed and developing countries [5,6,7]. However, the existence of socioeconomic barriers to access healthcare may compromise the achievement of the primary care goals established in public policies of health, potentially leading to deterioration in population health, wellbeing, and productivity [8].

The growing trends in public and private health expenditures due to chronic conditions in developing and developed countries emphasize the need for evidence on the connections between advances in PHC coverage in relation to population-level health outcomes, including hospitalization indicators, ensuring the adoption of evidence-based decision-making in public policies of health [9,10,11,12].

Recent studies showed the positive effects of access to PHC on hospitalization and mortality indicators, particularly in high-income countries, highlighting the role of preventive care, chronic disease monitoring, and timely referral to secondary care in the context of high-quality primary healthcare [10,12,13,14]. Furthermore, evidence shows that the focus on PHC strategies decrease the occurrence of hospitalizations, especially hospitalizations attributable to primary care-sensitive conditions [10,11,12,13,14], and that adequate PHC infrastructure and coverage reduces hospitalization expenditures [11].

Contrarily, low access to primary healthcare and multimorbidity may reduce the proper management of chronic diseases due to increase in self-medication and polypharmacy or a reduction in the use of medication, preventive, and screening services [15,16]. In addition, polypharmacy linked to multimorbidity, inadequate prescription, or self-medication in the absence of patients’ follow-up in the context of high-quality primary healthcare may aggregate further risks of hospitalization, especially among elderly individuals [17,18,19,20].

Although the access and quality of healthcare have been increasing in diverse population groups worldwide since the 1990s, substantial inequalities remain across social and economic development levels in several countries [21]. However, there is a lack of literature on the subject in developing countries, especially in Latin America [22,23,24]. Thus, the present study proposes a quantitative investigation on the connections between primary healthcare quality and effectiveness on hospitalization indicators, in addition to the identification of its effects on inequalities in hospitalizations within the Brazilian health system. Advances in universal healthcare coverage in Brazil, especially referring to the promotion of quality and effectiveness in primary healthcare strategies, have been associated with improvements in population health since the inception of the Brazilian Unified Health System (SUS) in 1988 [25,26,27].

The SUS provides healthcare to patients free of charge, being financed through taxes at the national, state, and local levels. Furthermore, diverse studies indicate a gradual reduction in socioeconomic inequalities in healthcare in Brazil in recent decades [25,28]. Yet, there is an absence of evidence at the population level on inequalities regarding the quality and effectiveness of healthcare within the Brazilian health system [25,26,28,29]. The lack of studies exploring the association between the dimensions of utilization, quality, and effectiveness represent a gap in research and management of national health systems in Latin American [30].

The study proposes to contribute with empirical elements to foster improvements in public policies of health in the country regarding equity in healthcare utilization, quality, and effectiveness in the context of primary healthcare and its associations with hospitalization indicators [27]. Therefore, considering the alignment of the principles of the Brazilian health system with global efforts to strengthen PHC strategies and reduce preventable hospitalizations, the following hypotheses were investigated in the study:

- Trends in the occurrence and frequency of hospitalizations, and length of stay presented declines in recent decades following the consolidation of advances in supply of healthcare through the SUS;
- Higher primary healthcare quality and effectiveness is linked to reductions in the occurrence and frequency of hospitalizations, and length of stay, whilst increasing the perception of quality of hospital care;
- Higher perception of primary healthcare quality and effectiveness is associated with lower inequalities in hospitalizations, inpatient days, and perception of quality of hospital care.

## 2. Materials and Methods

### 2.1. Study Design

The present study is based on the empirical analysis of individual-level information from cross-sectional surveys representative at the population level, executed by the Brazilian Institute for Geography and Statistics (IBGE) in 1998, 2003, 2008, 2013, and 2019.

### 2.2. Datasets

Three datasets of the National Household Sample Surveys (PNAD) from 1998, 2003, and 2008 and two datasets of the National Health Surveys (PNS) from 2013 and 2019 were combined into a single database to allow the investigation of associations between primary healthcare quality and effectiveness in relation to hospitalizations in Brazil. The PNAD is a cross-sectional annual survey on a sample of households selected using probabilistic process in three stages (municipalities, census tracts, and households), including supplementary information on health conditions and healthcare in 1998, 2003, and 2008. The PNS is a cross-sectional survey focusing on health and healthcare based on a sample of individuals selected through probabilistic process in three stages (census tracts, households, and individuals) performed in 2013 and 2019.

Information from the population census was used to estimate the representativeness of the survey samples and to plan the data collection through a selection of households and individuals to participate in the PNAD and PNS surveys. The PNAD and PNS surveys present several methodological similarities, including data representativeness and data collection tools. The demographic, socioeconomic, and health questionnaires of the surveys were applied by trained interviewers, being predominantly based on closed-ended questions.

The data collected in the surveys were organized into anonymized datasets and made publicly available on the IBGE website corresponding to PNAD (https://www.ibge.gov.br/estatisticas/sociais/populacao/19897-sintese-de-indicadores-pnad2.html?=&t=microdados, accessed on 3 March 2025) and PNS (https://www.ibge.gov.br/estatisticas/downloads-estatisticas.html, accessed on 3 March 2025). The present study included only individual-level data consistent across surveys, based on a selection of data reported by adult individuals (≥18 years old) through questions with similar phrasing and compatible response options.

Variables with substantial changes in the phrasing of the question or significant modifications in the response options were excluded from the datasets to avoid potential bias in the analyses. Only variables presenting minor changes in phrasing without compromising its interpretation (e.g., modifications in the code of the variable in the questionnaire and in the dataset or the division of one question with multiple options into multiple questions with binary responses) or minor modifications in response options (e.g., shuffle in order of alternatives or disaggregation of composite options into separate options) were included in the analyses.

### 2.3. Variables

The selection of variables from the survey datasets was based on the similarity of data across surveys, including demographic, socioeconomic, and health characteristics of the individuals; characteristics of households; and control variables referring to geographical characteristics and year of the survey (Table 1).

- Dependent variables:
  ○ Occurrence of hospitalization last year (binary: no or yes);
  ○ High frequency of hospitalization (binary: ≤3 times or >3 times during last year), considering mean readmissions among Brazilian adults in previous study at population level [31];
  ○ High length of stay (binary: ≤7 inpatient days or >7 inpatient days during last year), considering mean length of stay among Brazilian adults in previous study at the population level [31];
  ○ Perception of a good quality of hospital care (binary: no or yes).
- Independent variables:
  ○ Self-assessed health status (binary: less than good or good);
  ○ Mobility limitations (binary: no or yes);
  ○ Multimorbidity (binary: no or yes);
  ○ Type of hospital (binary: public or private);
  ○ Source of financing for hospitalization (three categorical variables referring to health insurance, out-of-pocket, and SUS: no or yes);
  ○ Dentist visit last year (binary: no or yes);
  ○ Use of primary healthcare in the last two weeks (binary: no or yes);
  ○ Perception of primary healthcare quality (binary: less than good, and good);
  ○ Primary healthcare effectiveness (continuous: proportion of days dedicated to solve health issues during the last two weeks).
- Control variables:
  ○ Area (binary: rural or urban);
  ○ State (27 categorical variables referring to 26 states and the federal capital: no or yes);
  ○ Year of the survey (five categorical variables referring to year of the PNAD and PNS surveys).
- Moderating variables:
  ○ Sex (binary: male or female);
  ○ Age (continuous: years);
  ○ Skin color/ethnicity (five categorical variables referring to black, brown, indigenous, white or yellow: no or yes);
  ○ Educational attainment (continuous: years of education);
  ○ Occupational status (binary: employed or unemployed);
  ○ Household size (discrete: individuals in the household);
  ○ Household income per capita in adult equivalents (continuous: international currency $ in 2022 purchase power parity, PPP);
  ○ Health insurance ownership (binary: no or yes).

Variables based on the self-declaration of individuals into predefined categories referred to: skin color/ethnicity, health status, use of health services, and hospitalization. The variables regarding skin color/ethnicity were based on five categories from traditional categorization adopted in Brazilian population surveys: Black, Brown, Indigenous, White, and Yellow. The variable of health status was based on individuals’ self-assessment of health.

Mobility limitations were declared by individuals, referring to difficulty in walking 100 m. The diagnosis of chronic diseases was based on the previous medical diagnosis of one of the following diseases listed in the questionnaire: orthopedic problems, arthritis, cancer, diabetes, asthma, hypertension, heart diseases, chronic kidney disease, depression, and tendinitis. Multimorbidity was based on the declaration of medical diagnosis of two or more of the aforementioned chronic diseases.

The use of health services was self-declared by individuals considering demand for healthcare in the last two weeks (primary healthcare utilization) or in the last year (dentist visit). The use of primary healthcare in the previous two weeks was also assessed regarding the perception of quality (less than good and good), whereas effectiveness was measured according to the number of days required to the resolution of the health issue, considering the period within the two weeks before the survey, i.e., fewer days needed to resolve health problems indicated higher healthcare effectiveness.

The hospitalization indicators encompassed the occurrence of hospitalizations in the last year, length of stay (inpatient days) in the last year, and individual’s perception of good quality of care in the hospitalization. However, the variable referring to perception of quality in the hospitalization was only available between 1998 and 2013; therefore, statistical analyses on quality of care in the hospital context exclude data from 2019. The source of funding for hospitalization was declared by individuals into three categories: health insurance, out-of-pocket disbursement, or government expenditures within SUS. The type of hospital was declared by individuals into two categories: public or private.

Household income per capita in adult equivalents was estimated through the division of the household income by adult equivalents in the household, based on the adult equivalent scale (*e_j_*) with 0.75 weight for individuals ≤ 14 years old (Equation (1)).

(1) $$e_{j}={\left(A_{j}+{\Phi .K}_{j}\right)}^{\theta }$$

where *A_j_* = adults in household *j*; *K_j_* = children ≤ 14 years old in household *j*; and *Φ* = *θ* = 0.75, weight defined in the literature on survey data [32]. The information was updated to December 2022 (period of reference), and converted into purchase power parity (PPP) to allow international comparisons, using the corresponding PPP conversion factor available at the World Bank website [33].

### 2.4. Statistical Analyses

Descriptive statistics were based on mean and standard error for continuous variables, and frequencies for categorical variables. Pairwise comparisons and marginal analyses were conducted to assess differences in patients’ perceptions of quality of care in hospitalization across types of hospitals and healthcare financing.

The estimation of logistic regression models was conducted to investigate factors associated with the probability of hospitalization, high frequency of hospitalizations, and high length of stay (inpatient days) in the previous year, in addition to the perception of quality of care in the hospitalization (outcome variables). The models focus particularly on the associations with primary healthcare quality and effectiveness (variables of interest), controlling for the sociodemographic, economic, and health characteristics of individuals and household characteristics (Equation (2)).

(2) $$\log\left(\frac{\pi_{ijt}}{1-\pi_{ijt}}\right)=\beta_{0}+\beta_{1}.S_{ijt}+\beta_{2}.H_{ijt}+\beta_{3}.{HH}_{jt}+\beta_{4}.C_{jt}$$

where *π_ijt_* = probability of hospitalization for individual *i* in household *j* in period *t*; *S_ijt_* = matrix of the demographic and socioeconomic characteristics of individual *i* in household *j* in period *t*; *H_ijt_* = matrix of the health characteristics of individual *i* in household *j* in period *t*; *HH_jt_* = matrix of the characteristics of household *j* in period *t*; *C_jt_* = matrix of control variables referring to the state of residence, year of the survey, and interaction between the state of residence and year of the survey. The statistical analyses were performed using the software Stata, version 17.0, adopting a significance level of *p* < 0.05.

Furthermore, the investigation of trends and factors associated with inequalities in hospitalization indicators was based on the estimation of concentration curves [34], which show the proportion of hospitalization indicators attributable to the cumulative proportion of individuals in the population, ordered according to the income level (Equation (3)).

(3) $$C=\frac{2\;}{n\mu }\;\sum_{i=1}^{N}y_{i}\;R_{i}-1$$

where *y_i_* = hospitalization indicator of interest in the analysis corresponding to the individual *i*; *µ* = mean of *y*; *R_i_* = classification of the *i*th individual in the income distribution; *N* = sample size.

The concentration indexes (CIs) corresponding to the concentration curves are estimated using similar equations to the Gini index [35], allowing to calculate inequalities according to individual’s income level (Equation (4)).

(4) $$CI=\frac{1}{\mu }\sum_{k}\left(\beta_{k\;}{\bar{x}}_{k}\right)C_{k}+\frac{GC}{\mu }$$

where *GC* = generalized concentration index for residual (*ε*) defined by $GC=\frac{2}{N}\sum_{i=1}^{N}\varepsilon_{i}R_{i}$; ${\bar{x}}_{k}$ = mean of *x_k_*.

The determinants of inequalities may be disaggregated through models estimating effects of individuals’ characteristics (*X*) and external factors (*W*) on hospitalization indicators [36], according to Equation (5).

(5) $$y*=\beta_{1}^{\prime }.X+\beta_{2}^{\prime }.W+\varepsilon$$

where y* = latent unobserved variable referring to the hospitalization indicators; *β_k_* = coefficient; ε = error term. The matrix *X* corresponds to the individuals’ characteristics influencing hospitalizations (age, sex, skin color/ethnicity, health status, physical limitations, and chronic diseases), and the matrix *W* includes variables linked to external factors (education, income, health insurance ownership, occupation, family characteristics, area, and state of residence). The models include only variables with coefficients with statistical significance *p* < 0.05 (Equation (5)).

### 2.5. Ethical Considerations

The study protocol was approved by the Brazilian National Research Ethics Commission (CONEP opinion #3.529.376, approval on 23 August 2019), following the ethical principles of the Helsinki Declaration. The present study was waived of a requirement to obtain informed consent due to use of publicly available datasets containing anonymized information from surveys conducted by the Brazilian Institute for Geography and Statistics (IBGE). The website of the IBGE presents public links to access anonymized datasets of the surveys: PNAD (https://www.ibge.gov.br/estatisticas/sociais/populacao/19897-sintese-de-indicadores-pnad2.html?=&t=microdados, accessed on 3 March 2025) and PNS (https://www.ibge.gov.br/estatisticas/downloads-estatisticas.html, accessed on 3 March 2025).

## 3. Results

Most individuals were female (52.61%) between 18 and 34 years old (39.21%) with basic educational attainment (8.72 years), self-declared as Brown (40.87%) or White (49.72%) skin color, employed (61.87%), and living in urban areas (85.05%). Trends show signs of demographic transition in Brazilian population, considering ageing of individuals (from 39.76 years old in 1998 to 44.83 years old in 2019) and a reduction in household residents (from 4.33 in 1998 to 3.30 in 2019), whereas educational attainment (from 6.97 years in 1998 to 9.99 years in 2019) and income level (from 714.53$ PPP in 1998 to 1081.68$ PPP in 2019) presented an increase throughout the period (Table 2).

Regarding health and healthcare characteristics, most individuals declared good health status (77.88%). The diagnosis of multimorbidity (from 21.52% in 1998 to 11.28% in 2019) showed decreasing trends, whilst there was increase in the occurrence of mobility limitations (from 1.37% to 3.30%) and dentist visits (from 32.09% to 48.84% in 2019) in recent decades. Most individuals with hospitalizations during the last year were financed through the public sector (60.42%) in public hospitals (63.53%), indicating a good quality of care in the hospitalization (99.73%). Similarly, individuals accessing primary healthcare in the last two weeks previous to the surveys indicated a perception of good quality (99.37%) and effectiveness (97.35%) in primary healthcare (Table 3).

The perception of good quality of care during hospitalizations was higher among individuals accessing private hospitals (significant differences varying between −0.020 in 1998 and −0.036 in 2019), financed through private funding sources (significant differences varying between −0.020 in 1998 and −0.033 in 2019), i.e., health insurance or out-of-pocket payment (Table 4).

The occurrence of hospitalizations was inversely associated with effectiveness in primary healthcare (OR = 0.980), being female (OR = 0.948), educational attainment (OR = 0.991), declaring good health status (OR = 0.712), dentist visits during the last year (OR = 0.812), and household income per capita (OR = 0.967), whereas it was positively associated with age (OR = 1.003), mobility limitations (OR = 1.628), diagnosis of multimorbidity (OR = 1.311), and health insurance ownership (OR = 1.273) (Table 5).

Similarly, a high frequency of hospitalizations (>3 hospitalizations within the previous year) and high length of stay (>7 inpatient days in the last year) were negatively associated with primary healthcare effectiveness (OR = 0.980 and OR = 0.992, respectively), declaring a good health status (OR = 0.688 and OR = 0.598, respectively), and household income per capita (OR = 0.954 and OR = 0.970, respectively); whilst positively associated with age (OR = 1.006 and OR = 1.014, respectively), and mobility limitations (OR = 1.550 and OR = 1.411, respectively) (Table 6).

The assessment of good quality of care during hospitalizations was positively associated with the perception of good quality of care in primary healthcare (OR = 10.311), age (OR = 1.016), good health status (OR = 1.421), financing through health insurance (OR = 1.851), and living in rural areas (OR = 1.424). Individuals diagnosed with multimorbidity (OR = 0.748) and individuals accessing public hospitals (OR = 0.655) perceived a lower quality of care during hospitalization (Table 7).

Trends in concentration indexes of hospitalizations and inpatient days showed a decrease in inequalities favoring individuals with lower income levels from 1998 to 2013, whereas concentration indexes referring to the perception of quality of hospital care indicated a lower level of inequality favoring individuals with higher income levels (Table 8).

**Figure 1:**
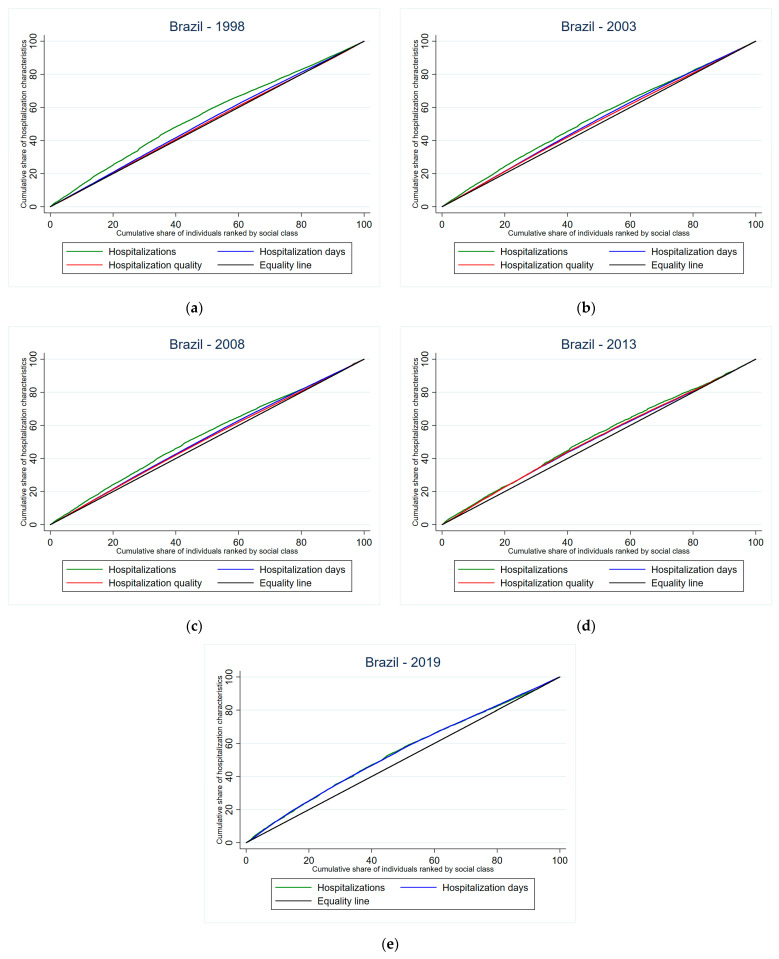
Concentration curves of inequalities in hospitalizations, inpatient days, and quality of hospital care ^§^ in Brazil: (**a**) 1998; (**b**) 2003; (**c**) 2008; (**d**) 2013; (**e**) 2019. ^§^ assessment of quality of care in hospitalizations included in the surveys of PNAD 1998, 2003, and 2008 and PNS 2013.

The concentration curves show the graphical representation of the concentration indexes estimated for hospitalizations, inpatient days, and perception of quality of hospital care according to year of the survey (Figure 1).

**Figure 2:**
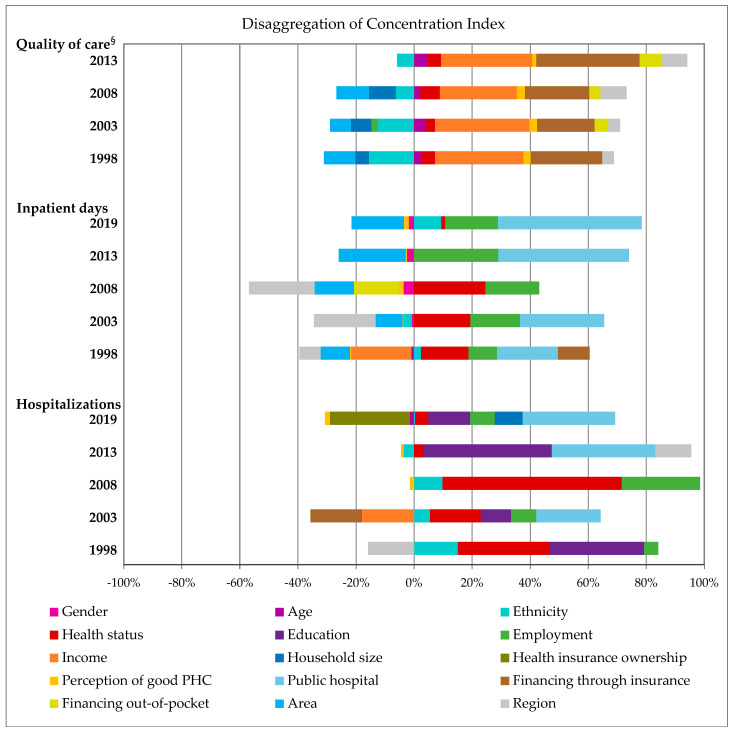
Disaggregation of concentration indexes for hospitalizations, inpatient days, and quality of hospital care, according to year of survey. Brazil, 1998–2019 ^§^. ^§^ assessment of quality of care in hospitalizations included in the surveys of PNAD 1998, 2003, and 2008 and PNS 2013; PHC = primary healthcare.

The major factors linked to inequalities in hospitalization indicators in Brazil were identified through the disaggregation of concentration indexes (Figure 2). Household income and financing hospitalizations through health insurance contributed to inequalities in the perception of quality of hospital care towards high-income individuals, whereas skin color/ethnicity contributed to inequalities favoring low-income individuals.

Similarly, access to public hospitals, employment, and good health status were associated with inequalities in length of stay and hospitalizations favoring low-income individuals. However, geographical characteristics (region and area) contributed to inequalities in inpatient days towards high-income individuals, whilst educational attainment was linked to inequalities in hospitalizations towards low-income individuals (Figure 2).

Patients’ perceptions on primary healthcare quality represented minor influence in inequalities regarding hospitalizations and length of stay favoring high-income individuals; although also contributing to inequalities in the assessment of quality of hospital care towards high-income individuals (Figure 2).

## 4. Discussion

The study investigated associations between primary healthcare quality and effectiveness in relation to hospitalization indicators in Brazil from 1998 to 2019. The findings allowed to confirm the first hypothesis of the study, showing that there was a decline in the occurrence and frequency of hospitalizations and the length of stay during the period from 1998 to 2013, which marked consolidation of advances in supply of healthcare through the SUS [26,27,28,29,30].

Furthermore, the second hypothesis of the study was confirmed through logistic regression models indicating that a higher primary healthcare effectiveness was associated with a reduction in the occurrence of hospitalizations and length of stay, similar to evidence from high-income countries [9,10,11,12]. In addition, higher primary healthcare quality was linked to a higher perception of quality of hospital care, in accordance with studies on primary healthcare strategies for the improvement in health system outcomes [37].

The third hypothesis was partially supported by the results obtained in the estimation of concentration indexes, since only patients’ perceptions of primary healthcare quality were associated with inequalities in hospitalizations, inpatient days, and perception of quality of hospital care. Primary healthcare effectiveness presented a lack of significance in the estimation of concentration indexes; yet, a previous study showed an association between the perception of primary healthcare quality and effectiveness in the Brazilian health system [38].

Primary healthcare quality was associated with the perception of higher quality of hospital care; however, contrarily to the evidence in the literature [39,40], patients’ income showed a lack of significance. Multimorbidity was positively associated with the occurrence of hospitalization, in accordance to evidence from previous studies in UK and China [41,42]. However, the diagnosis of multiple chronic conditions was also negatively associated with the perception of quality of hospital care, contrarily to the findings from a study conducted in Australia [9].

Trends in hospitalization indicators showed a reduction in inequalities towards low-income individuals from 1998 to 2013, which is supported by previous evidence from the literature on the consolidation of universal healthcare coverage in Brazil until 2008 [26,27,28,29]. Yet, primary healthcare quality presented a minor influence on inequalities in hospitalizations, inpatient days, and perception of quality of hospital care.

The study has limitations regarding study design and data collection. The use of cross-sectional data hinders the establishment of potential causal relations through the statistical analysis. Nevertheless, considering the availability of individual-level datasets selected through complex sampling procedures, the study allowed the identification of connections between advances in primary healthcare quality and effectiveness in relation to hospitalization indicators, showing results representative at the population level.

Furthermore, the data collection tools adopted by the Brazilian Institute for Geography and Statistics were based on self-reported information regarding health status and healthcare utilization, which may contain bias. However, using robust empirical strategies with control variables for the state of residence, year of the survey, and interaction between state of residence and year of the survey allowed to obtain valid estimates to inform public health decision-making processes, thus minimizing potential errors.

Finally, it is important to emphasize that the study presents substantial contributions to the field of knowledge, considering the absence of evidence on associations between primary healthcare quality and effectiveness in relation to hospitalization indicators in developing countries, particularly in Latin American countries [43,44]. Therefore, the findings of the study align with global priorities linked to population ageing towards the reduction in hospitalizations attributable to multimorbidity and primary care-sensitive conditions through strengthening PHC strategies, including efforts for the promotion of integrated care within health systems based on universal health coverage in Brazil and other countries [16,17,18,19,20,45,46,47,48].

## 5. Conclusions

The findings of the study contribute to advances in the field of knowledge, bridging the gap in the literature regarding connections between PHC and hospitalizations in Latin American countries. Trends in hospitalization indicators identified in the present study were associated with the consolidation of the SUS via advances in universal healthcare coverage at the primary level in Brazil in recent decades. The adoption of strategies of health promotion and disease prevention at the primary care level have been shown to reduce public and private expenditures in health in diverse countries, through the promotion of efficiency, quality, and effectiveness in national health systems. Additionally, the study highlighted the simultaneous effects of demographic and epidemiological transitions on hospitalization indicators, emphasizing the strategic role of PHC in monitoring multimorbidity and reducing preventable hospitalizations attributable to primary care-sensitive conditions in the context of ageing populations.

Policies designed to foster improvements in primary healthcare quality and effectiveness in Brazil presented effects in indicators referring to the occurrence and frequency of hospitalizations, length of stay, and perception of quality of hospital care throughout the period from 1998 to 2013. Recent changes in health policies, particularly referring to primary healthcare financing and incentives related to payment for performance, may represent throwbacks in universal health coverage, potentially affecting population health, wellbeing, and productivity in the next years.

Additional studies focusing on associations between policies for the promotion of primary healthcare, hospitalization, and health outcomes at the population level should investigate other attributes of national health systems in developing countries, particularly in Latin America, considering the absence of literature on the subject in the region. Future research will focus on the causes for hospitalization and procedures performed during hospitalizations in Brazil, allowing the further exploration of connections among population dynamics, epidemiological transition, and health system organization features in relation to the policy-level characteristics of the national health system.

## Figures and Tables

**Table 1:** Characteristics of the variables in the dataset. Brazil, 1998–2019.

Variable	N	μ	SD	Min	Max
Sex	1,097,786	0.53	0.50	0	1
Age	1,097,681	41.14	16.68	18	113
Skin color/ethnicity					
Black	1,097,566	0.08	0.27	0	1
Brown	1,097,566	0.45	0.50	0	1
Indigenous	1,097,566	0.00	0.06	0	1
White	1,097,566	0.46	0.50	0	1
Yellow	1,097,566	0.01	0.07	0	1
Educational attainment	1,045,367	8.47	4.91	0	18
Employed	1,079,732	0.62	0.49	0	1
Household size	1,128,969	3.84	1.95	1	27
Household income per capita	1,107,597	783.50	1326.23	0.00	131,645.00
Health insurance ownership	1,097,740	0.26	0.44	0	1
Good health status	1,128,969	0.76	0.43	0	1
Mobility limitations	1,128,969	0.02	0.13	0	1
Multimorbidity	1,128,969	0.15	0.36	0	1
Dentist visit last year	1,097,749	0.39	0.49	0	1
PHC last two weeks	1,097,749	0.16	0.36	0	1
Perception of good PHC quality	1,128,969	0.99	0.08	0	1
PHC effectiveness	167,969	96.81	8.05	7.14	100.00
Hospitalization last year	1,097,730	0.08	0.27	0	1
High frequency of hospitalization	83,974	0.04	0.20	0	1
High length of stay	1,128,403	0.01	0.11	0	1
Type of hospital					
Public hospital	84,000	0.65	0.48	0	1
Private hospital	84,000	0.34	0.48	0	1
Hospitalization financing					
Health insurance	84,000	0.26	0.44	0	1
Out-of-pocket	84,000	0.12	0.33	0	1
SUS	84,000	0.66	0.48	0	1
Perception of good quality of care	906,780	1.00	0.05	0	1
Area	1,128,969	0.17	0.37	0	1
State	1,128,969	31.42	10.93	11	53
Year	1,128,969	2008	7	1998	2019

N = observations; μ = mean; SD = standard deviation; Min = minimum; Max = maximum.

**Table 2:** Personal characteristics of individuals, according to year of the survey. Brazil, 1998–2019.

Characteristics	1998	2003	2008	2013	2019	Total
Sex						
Male	47.92	47.73	47.68	47.10	46.84	47.39
Female	52.08	52.27	52.32	52.90	53.16	52.61
Age *	39.76	40.19	41.45	42.85	44.83	42.09
	0.17	0.06	0.06	0.10	0.09	0.04
Skin color/ethnicity						
Black	6.16	6.42	7.55	9.14	11.10	8.35
Brown	36.63	39.01	41.50	42.13	43.24	40.87
Indigenous	0.21	0.20	0.30	0.42	0.49	0.34
White	56.36	53.87	49.99	47.44	44.30	49.72
Yellow	0.65	0.52	0.67	0.87	0.87	0.73
Educational attainment *	6.97	7.79	8.60	9.44	9.99	8.72
	0.20	0.05	0.04	0.05	0.04	0.02
Occupational status						
Employed	62.80	62.18	64.77	61.35	58.98	61.87
Unemployed	37.20	37.82	35.23	38.65	41.02	38.13
Household size *	4.33	4.08	3.79	3.56	3.30	3.76
	0.04	0.01	0.01	0.01	0.01	0.01
Household income per capita *	714.53	658.50	801.24	1047.22	1081.68	887.01
	52.86	8.51	9.12	22.64	16.62	9.11
Area						
Urban	81.42	85.45	84.65	86.33	86.21	85.05
Rural	18.58	14.55	15.35	13.67	13.79	14.95

* mean and standard error.

**Table 3:** Health and healthcare characteristics of individuals, according to the year of the survey. Brazil, 1998–2019.

Characteristics	1998	2003	2008	2013	2019	Total
Health insurance ownership	26.63	26.88	28.11	29.72	26.98	27.74
Good health status	71.62	72.37	71.32	85.39	84.58	77.88
Mobility limitations	1.37	1.34	1.72	2.45	3.30	2.14
Multimorbidity	21.52	18.12	18.47	8.80	11.28	14.99
Dentist visit	32.09	37.60	39.00	44.13	48.84	41.21
PHC utilization	14.34	15.81	15.67	16.61	19.84	16.71
PHC effectiveness *	95.86	95.88	96.16	99.54	98.15	97.35
	0.09	0.06	0.06	0.04	0.06	0.04
Perception of good PHC quality	99.65	99.60	99.51	99.71	98.61	99.37
Occurrence of hospitalization	8.43	8.05	8.03	6.61	7.20	7.58
High frequency of hospitalization	3.59	3.77	4.06	4.69	3.72	3.97
High length of stay	1.44	1.32	1.35	1.14	1.30	1.30
Type of hospital						
Public	59.56	61.12	67.32	65.12	63.70	63.56
Private	39.82	38.41	32.51	34.51	36.01	36.07
Hospitalization financing						
Health insurance	25.91	25.89	26.59	29.07	30.18	27.65
Out-of-pocket	14.82	10.85	11.12	11.74	11.62	11.93
SUS	59.27	63.26	62.29	59.19	58.20	60.42
Perception of good quality of care ^§^	99.76	99.78	99.71	99.67		99.73

* mean and standard error; ^§^ assessment of quality of care in hospitalizations included in the surveys of PNAD 1998, 2003, and 2008 and PNS 2013.

**Table 4:** Pairwise comparisons and marginal predictions for perception of quality of care in hospitalization according to type of hospital and source of funding. Brazil, 1998–2013 ^§^.

Perception of Good Quality of Care		1998	2003	2008	2013
Public hospital	Margin	0.964	0.966	0.955	0.938
	SE	0.002	0.002	0.002	0.004
Private hospital	Margin	0.983	0.985	0.983	0.974
	SE	0.002	0.001	0.002	0.004
Pairwise comparison	Contrast	−0.020 *	−0.020 *	−0.028 *	−0.036 *
	SE	0.003	0.002	0.002	0.006
SUS funding	Margin	0.964	0.966	0.955	0.939
	SE	0.002	0.002	0.002	0.004
Private funding	Margin	0.984	0.987	0.982	0.972
	SE	0.002	0.002	0.002	0.004
Pairwise comparison	Contrast	−0.020 *	−0.020 *	−0.026 *	−0.033 *
	SE	0.003	0.002	0.003	0.006

^§^ assessment of quality of care in hospitalizations included in the surveys of PNAD 1998, 2003, and 2008 and PNS 2013; SE = standard error; * *p* < 0.001.

**Table 5:** Odds ratios of logistic regression model referring to the occurrence of hospitalization. Brazil, 1998–2019.

Variables		Hospitalization			
		OR	SE	Sig.	95% CI
PHC effectiveness	(%)	0.980	0.001	***	0.98; 0.98
Sex	(fem = 1)	0.948	0.021	*	0.91; 0.99
Age	(years)	1.003	0.001	***	1.00; 1.00
Educational attainment	(years)	0.991	0.003	**	0.99; 1.00
Good health status	(yes = 1)	0.712	0.018	***	0.68; 0.75
Mobility limitations	(yes = 1)	1.628	0.083	***	1.47; 1.80
Multimorbidity	(yes = 1)	1.311	0.035	***	1.24; 1.38
Health insurance ownership	(yes = 1)	1.273	0.036	***	1.20; 1.35
Dentist visit	(yes = 1)	0.812	0.020	***	0.77; 0.85
Household income per capita	(ln)	0.967	0.005	***	0.96; 0.98
Area	(rural = 1)	1.021	0.030		0.96; 1.08

OR = odds ratio; SE = standard error; 95% CI = 95% confidence interval; PHC = primary healthcare; *** *p* < 0.001; ** *p* < 0.01; * *p* < 0.05. Model includes control variables for state of residence, year of the survey, and interaction between state of residence and year of the survey.

**Table 6:** Odds ratios of logistic regression models referring to frequency of hospitalization and length of stay. Brazil, 1998–2019.

Variables		High Frequency of Hospitalization				High Length of Stay			
		OR	SE	Sig.	95% CI	OR	SE	Sig.	95% CI
PHC effectiveness	(%)	0.980	0.002	***	0.98; 0.98	0.992	0.002	***	0.99; 1.00
Sex	(fem = 1)	0.960	0.073		0.83; 1.11	0.620	0.031	***	0.56; 0.68
Age	(years)	1.006	0.002	**	1.00; 1.01	1.014	0.002	***	1.01; 1.02
Educational attainment	(years)	0.957	0.011	***	0.94; 0.98	1.001	0.006		0.99; 1.01
Good health status	(yes = 1)	0.688	0.069	***	0.57; 0.84	0.598	0.036	***	0.53; 0.67
Mobility limitations	(yes = 1)	1.550	0.197	**	1.21; 1.99	1.411	0.127	***	1.18; 1.68
Multimorbidity	(yes = 1)	1.212	0.107	*	1.02; 1.44	0.970	0.054		0.87; 1.08
Health insurance ownership	(yes = 1)	0.829	0.084		0.68; 1.01	0.738	0.047	***	0.65; 0.84
Dentist visit	(yes = 1)	0.851	0.078		0.71; 1.02	0.798	0.044	***	0.72; 0.89
Household income per capita	(ln)	0.954	0.019	*	0.92; 0.99	0.970	0.013	*	0.95; 0.99
Area	(rural = 1)	0.880	0.093		0.71; 1.08	0.743	0.047	***	0.66; 0.84

OR = odds ratio; SE = standard error; 95% CI = 95% confidence interval; PHC = primary healthcare; *** *p* < 0.001; ** *p* < 0.01; * *p* < 0.05. Model includes control variables for state of residence, year of the survey, and interaction between state of residence and year of the survey.

**Table 7:** Odds ratios of logistic regression model referring to the perception of good quality of care in hospitalization. Brazil, 1998–2013 ^§^.

Variables		Good Quality of Care			
		OR	SE	Sig.	95% CI
Perception of good PHC quality	(yes = 1)	10.311	1.174	***	8.25; 12.89
Sex	(fem = 1)	1.110	0.069		0.98; 1.25
Age	(years)	1.016	0.002	***	1.01; 1.02
Educational attainment	(years)	0.985	0.008		0.97; 1.00
Good health status	(yes = 1)	1.421	0.100	***	1.24; 1.63
Mobility limitations	(yes = 1)	1.293	0.182		0.98; 1.70
Multimorbidity	(yes = 1)	0.748	0.056	***	0.65; 0.87
Public hospital	(yes = 1)	0.655	0.074	***	0.53; 0.82
Financing through health insurance	(yes = 1)	1.851	0.239	***	1.44; 2.38
Financing out-of-pocket	(yes = 1)	1.211	0.149		0.95; 1.54
Household income per capita	(ln)	1.006	0.015		0.98; 1.04
Area	(rural = 1)	1.424	0.117	***	1.21; 1.67

^§^ assessment of quality of care in hospitalizations included in the surveys of PNAD 1998, 2003, 2008 and PNS 2013; OR = odds ratio; SE = standard error; 95% CI = 95% confidence interval; PHC = primary healthcare; *** *p* < 0.001. Model includes control variables for state of residence, year of the survey, and interaction between state of residence and year of the survey.

**Table 8:** Concentration indexes and horizontal inequality indexes in hospitalizations, inpatient days, and perception of quality of hospital care, according to year of the survey. Brazil, 1998–2019 ^§^.

**Hospitalizations**	**1998**	**2003**	**2008**	**2013**	**2019**
Concentration Index	−0.024	−0.014	−0.015	−0.009	−0.030
Horizontal Inequality	−0.008	−0.004	−0.007	−0.009	−0.028
**Inpatient days**	**1998**	**2003**	**2008**	**2013**	**2019**
Concentration Index	−0.082	−0.041	−0.044	−0.036	−0.046
Horizontal Inequality	0.155	0.135	0.108	−0.068	0.073
**Quality of care ^§^**	**1998**	**2003**	**2008**	**2013**	**2019**
Concentration Index	0.0004	0.0003	0.0005	0.0003	-
Horizontal Inequality	0.0013	0.0009	0.0002	−0.0001	-

^§^ assessment of quality of care in hospitalizations included in the surveys of PNAD 1998, 2003, and 2008 and PNS 2013.

## Data Availability

Datasets in the present study are publicly available on the platform of the Brazilian Institute for Geography and Statistics (*Instituto Brasileiro de Geografia e Estatística*, IBGE): PNAD (https://www.ibge.gov.br/estatisticas/sociais/populacao/19897-sintese-de-indicadores-pnad2.html?=&t=microdados, accessed on 3 March 2025) and PNS (https://www.ibge.gov.br/estatisticas/downloads-estatisticas.html, accessed on 3 March 2025).
